# Supplementary material for: Molecular tension indicators reveal unexpectedly complex regulation of tension in live mouse organs
Source: Commun Biol. 2026 Feb 19;9:455. doi: 10.1038/s42003-026-09746-0 (PMC13031782; doi:10.1038/s42003-026-09746-0)

Supplementary Information

Molecular tension indicators reveal unexpectedly complex regulation of tension in live mouse organs

Keita Fujiwara [1, 2], Katsunori Fujiki [3], Tomoya O Akama [1], Katsuhiko Shirahige [3, 4], Ichiro Shiojima [2], Tomoyuki Nakamura [1], *Maretoshi Hirai [1]

1. Department of Pharmacology, Kansai Medical University, Hirakata, Osaka 573-1010, Japan
2. Department of Medicine II, Kansai Medical University, Hirakata, Osaka 573-1010, Japan
3. Laboratory of Genome Structure and Function, Institute for Quantitative Biosciences, The University of Tokyo, Tokyo 113-0032, Japan
4. Laboratory of Chromosome dynamics and genome stability, Department of Cell and Molecular Biology, Karolinska Institutet, Stockholm 17177, Sweden

*Correspondence: Maretoshi Hirai

Email: hirai.mar@kmu.ac.jp (M.H.), telephone: +81-72-804-2368

This PDF file includes:

Supplementary Figures 1 to 5

Supplementary Figure legends

Supplementary Movie legends

Original blot/gel images

Other supplementary materials for this manuscript include the following:

Supplementary Movies 1 to 6

Supplementary Data 1 (.xlsx)

Supplementary Data 2 (.txt)

Supplementary Figure 1

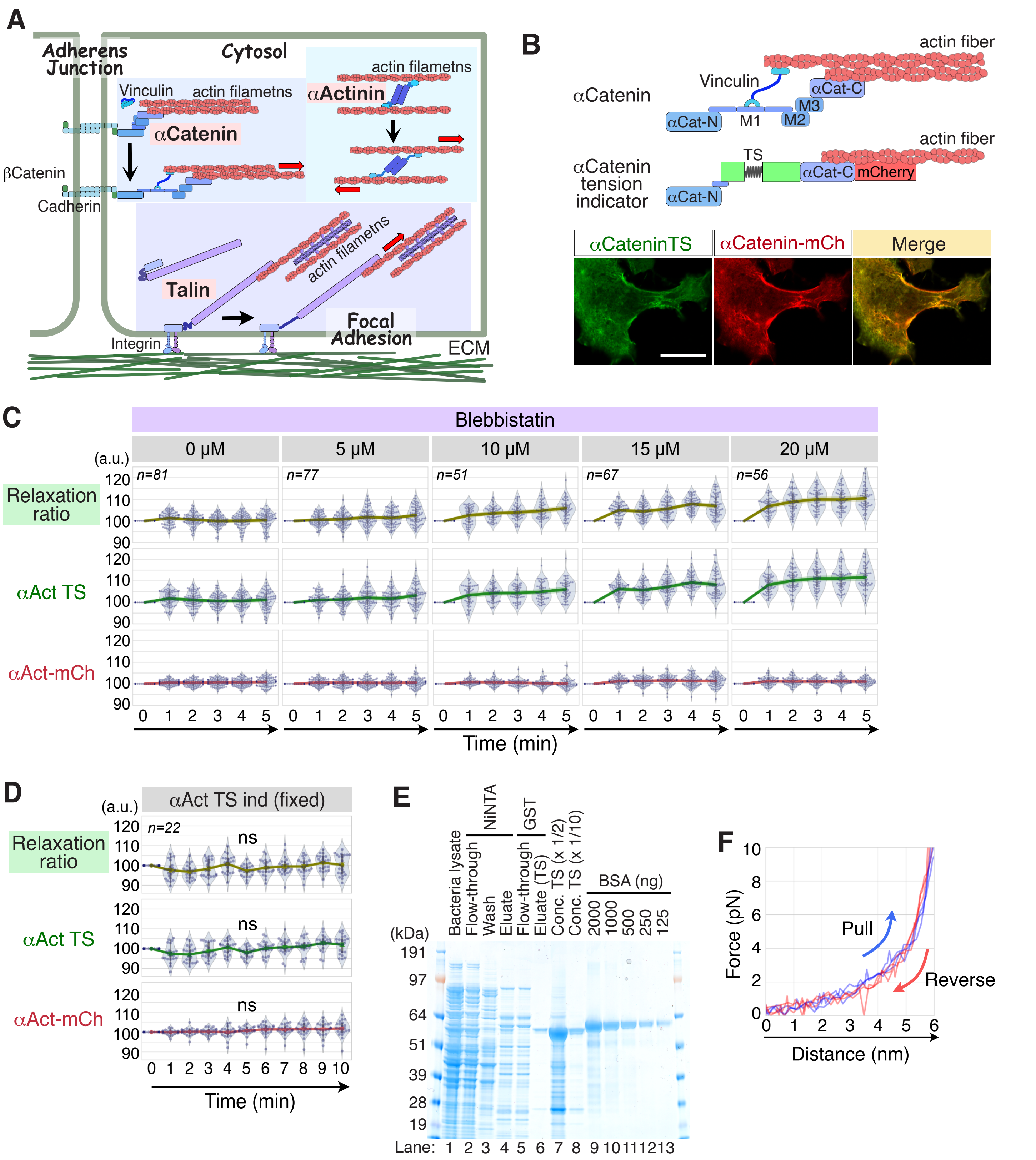

Supplementary Figure 1. **Development of the αActinin and αCatenin tension indicators. A,** Schematic illustration of cytoskeletal molecules involved in tension sensing, such as αCatenin, αActinin, Talin, and their interactions with actin filaments. These molecules have been applied to develop tension sensors (TSs) in the past. **B,** Schematic illustration of the domain structure of αCatenin under tension and the αCatenin tension indicator developed in this study. The M1, M2, and M3 domains of αCatenin are folded in the absence of tension. For the αCatenin tension indicator, the TS module was inserted between M1 and the C-terminal domain of αCatenin. (Bottom): Confocal fluorescence microscopy of NIH3T3 cells expressing the αCatenin tension indicator, which localizes at cell margins. Scale bar: 10 μm. **C,** Scatter and violin plots of quantification of time-lapse fluorescence microscopy of MDCKII cells expressing the αActinin tension indicator before and after the addition of different concentrations of blebbistatin. **D,** Scatter and violin plots of fixed MDCKII cells expressing the αActinin tension indicator, imaged under the same laser power and acquisition conditions as in (**C**) to assess photobleaching. Despite time-lapse imaging under identical settings, no appreciable photobleaching was observed. **E,** Coomassie blue staining of purified TS protein. Concentrated TS protein (Lane 7 and 8) was subjected to oligo-arm attachment. **F,** Force–distance curves of a single TS molecule during repeated loading–unloading cycles using optical tweezers. Blue and red traces indicate the pull and reverse phases, respectively. Nearly identical curves across cycles demonstrate the highly reversible mechanical response of the TS.

Supplementary Figure 1. **Development of the αActinin and αCatenin tension indicators. A,** Schematic illustration of cytoskeletal molecules involved in tension sensing, such as αCatenin, αActinin, Talin, and their interactions with actin filaments. These molecules have been applied to develop tension sensors (TSs) in the past. **B,** Schematic illustration of the domain structure of αCatenin under tension and the αCatenin tension indicator developed in this study. The M1, M2, and M3 domains of αCatenin are folded in the absence of tension. For the αCatenin tension indicator, the TS module was inserted between M1 and the C-terminal domain of αCatenin. (Bottom): Confocal fluorescence microscopy of NIH3T3 cells expressing the αCatenin tension indicator, which localizes at cell margins. Scale bar: 10 μm. **C,** Scatter and violin plots of quantification of time-lapse fluorescence microscopy of MDCKII cells expressing the αActinin tension indicator before and after the addition of different concentrations of blebbistatin. **D,** Scatter and violin plots of fixed MDCKII cells expressing the αActinin tension indicator, imaged under the same laser power and acquisition conditions as in (**C**) to assess photobleaching. Despite time-lapse imaging under identical settings, no appreciable photobleaching was observed. **E,** Coomassie blue staining of purified TS protein. Concentrated TS protein (Lane 7 and 8) was subjected to oligo-arm attachment. **F,** Force–distance curves of a single TS molecule during repeated loading–unloading cycles using optical tweezers. Blue and red traces indicate the pull and reverse phases, respectively. Nearly identical curves across cycles demonstrate the highly reversible mechanical response of the TS.

Supplementary Figure 2

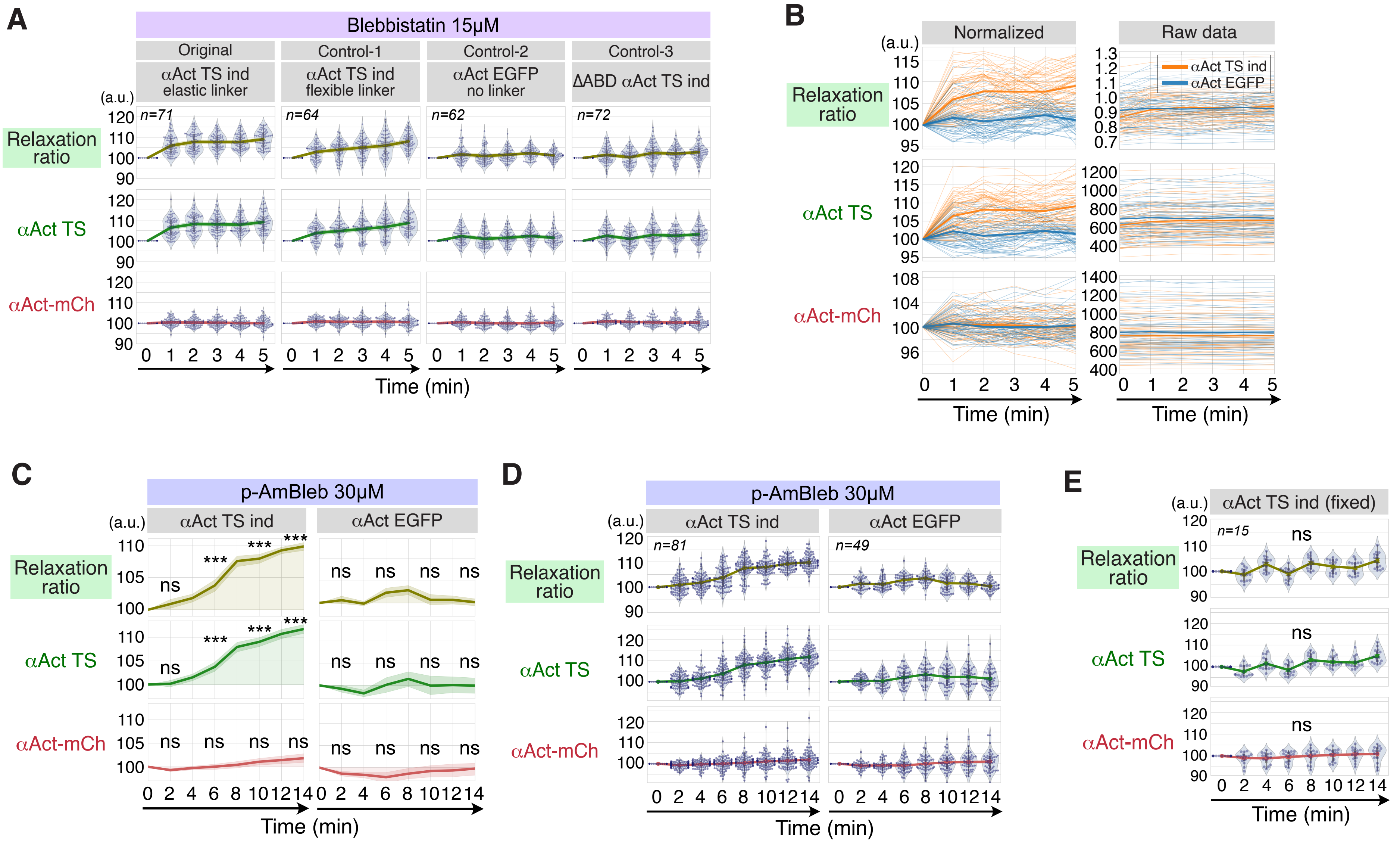

Supplementary Figure 2: **Validation of the αActinin tension indicator**. **A,** Scatter and violin plots of quantification of time-lapse fluorescence microscopy of MDCKII cells expressing the αActinin TS indicator and control constructs after the addition of 15 μM blebbistatin. The TS indicator exhibited a clear increase in relaxation ratio, whereas the controls showed reduced or minimal responses. **B,** Example of the normalization procedure applied to raw fluorescence intensities (green and red channels) for MDCKII cells expressing either αActinin TS indicator or αActinin-EGFP control. Left: line plots of normalized intensities; Right: raw fluorescence intensities prior to normalization. **C, D,** Line plots (**C**) and scatter/violin plots (**D**) showing quantification of time-lapse fluorescence microscopy of MDCKII cells expressing the αActinin TS indicator and αActinin-EGFP control after addition of 30 μM p-AmBleb. The αActinin TS indicator showed a marked increase in relaxation ratio, whereas αActinin-EGFP control showed little or no change. **E,** Photobleaching control using fixed MDCKII cells expressing the αActinin TS indicator. No appreciable fluorescence change was observed under the same conditions used in **(C)**. Each dot indicates the fluorescence intensity of each cell (**A, C, D, and E**). The shaded areas indicate the 95% confidence intervals (CIs) (**C**). Statistical analysis was performed using a one-way ANOVA, followed by the Scheffé post hoc test (**C**); ns: $p > 0.05$, *: $p < 0.05$, **: $p < 0.01$, ***: $p < 0.001$. Asterisks indicate statistical significance at 2, 6, 10, and 14 min compared to 0 min.

Supplementary Figure 2: **Validation of the αActinin tension indicator**. **A,** Scatter and violin plots of quantification of time-lapse fluorescence microscopy of MDCKII cells expressing the αActinin TS indicator and control constructs after the addition of 15 μM blebbistatin. The TS indicator exhibited a clear increase in relaxation ratio, whereas the controls showed reduced or minimal responses. **B,** Example of the normalization procedure applied to raw fluorescence intensities (green and red channels) for MDCKII cells expressing either αActinin TS indicator or αActinin-EGFP control. Left: line plots of normalized intensities; Right: raw fluorescence intensities prior to normalization. **C, D,** Line plots (**C**) and scatter/violin plots (**D**) showing quantification of time-lapse fluorescence microscopy of MDCKII cells expressing the αActinin TS indicator and αActinin-EGFP control after addition of 30 μM p-AmBleb. The αActinin TS indicator showed a marked increase in relaxation ratio, whereas αActinin-EGFP control showed little or no change. **E,** Photobleaching control using fixed MDCKII cells expressing the αActinin TS indicator. No appreciable fluorescence change was observed under the same conditions used in **(C)**. Each dot indicates the fluorescence intensity of each cell (**A, C, D, and E**). The shaded areas indicate the 95% confidence intervals (CIs) (**C**). Statistical analysis was performed using a one-way ANOVA, followed by the Scheffé post hoc test (**C**); ns: $p > 0.05$, *: $p < 0.05$, **: $p < 0.01$, ***: $p < 0.001$. Asterisks indicate statistical significance at 2, 6, 10, and 14 min compared to 0 min.

Supplementary Figure 3

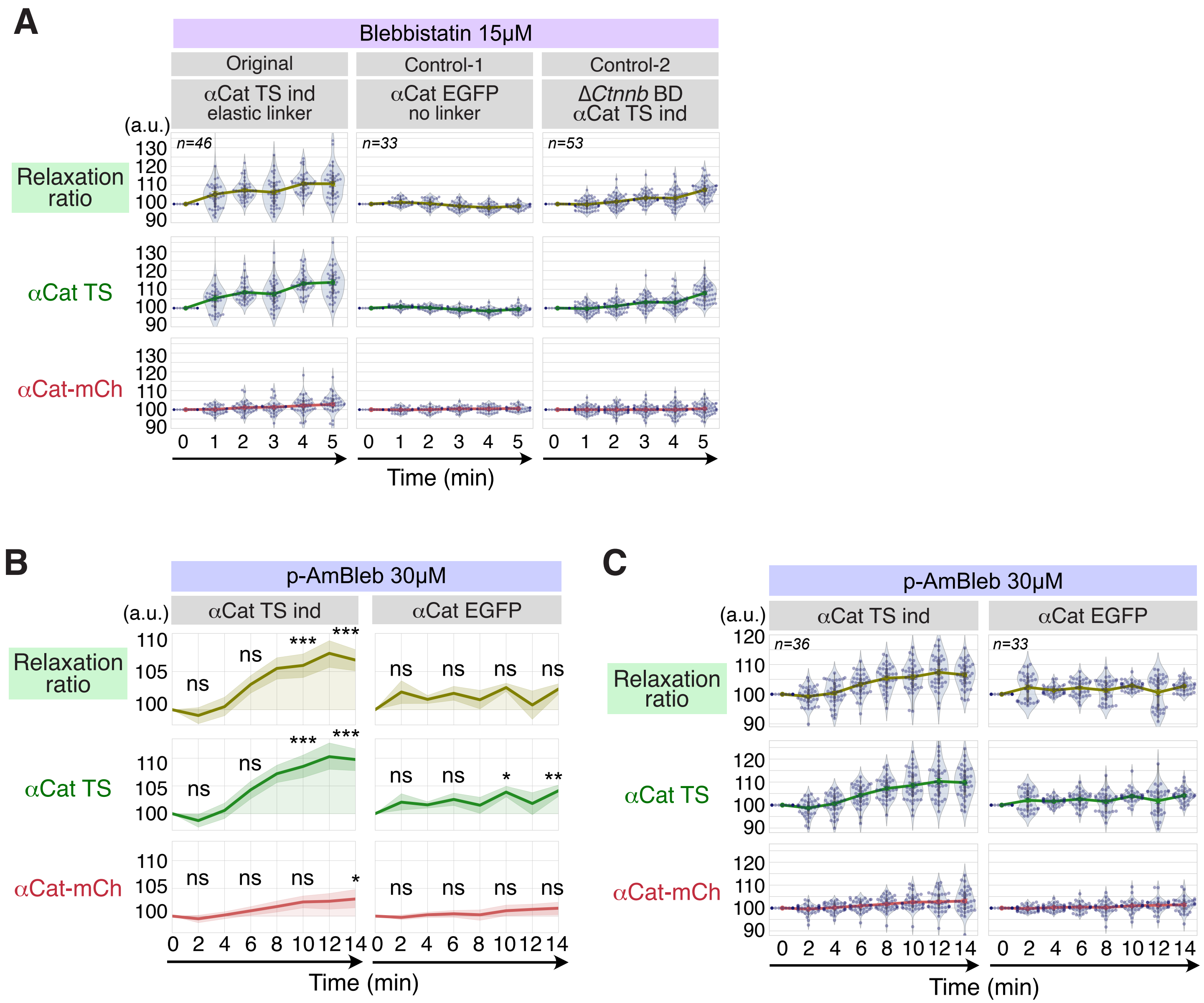

Supplementary Figure 3: **Validation of the αCatenin tension indicator**. **A,** Scatter/violin plots of time-lapse fluorescence microscopy of NIH3T3 cells expressing the αCatenin TS indicator and control constructs after the addition of 15 μM blebbistatin. The TS indicator showed a clear increase in relaxation ratio, whereas Control-1 (αCat EGFP lacking the TS linker) and Control-2 (Δ*Ctnnb* BD αCat TS lacking the β-catenin-binding domain) exhibited reduced or minimal responses. **B, C,** Quantification of NIH3T3 cells expressing the αCatenin TS indicator or αCatenin-EGFP control after the addition of 30 μM p-AmBleb. The αCatenin TS indicator showed a marked increase in relaxation ratio, whereas αCatenin-EGFP control showed little or no change. Each dot in the scatter plot indicates the fluorescence intensity of each cell (**A, C**). The shaded areas indicate the 95% CIs (**B**). Statistical analysis was performed using one-way ANOVA followed by the Scheffé post hoc test (**B**); ns: $p > 0.05$, *: $p < 0.05$, **: $p < 0.01$, ***: $p < 0.001$. Asterisks indicate statistical significance at 2, 6, 10, and 14 min compared to 0 min.

Supplementary Figure 3: **Validation of the αCatenin tension indicator**. **A,** Scatter/violin plots of time-lapse fluorescence microscopy of NIH3T3 cells expressing the αCatenin TS indicator and control constructs after the addition of 15 μM blebbistatin. The TS indicator showed a clear increase in relaxation ratio, whereas Control-1 (αCat EGFP lacking the TS linker) and Control-2 (Δ*Ctnnb* BD αCat TS lacking the β-catenin-binding domain) exhibited reduced or minimal responses. **B, C,** Quantification of NIH3T3 cells expressing the αCatenin TS indicator or αCatenin-EGFP control after the addition of 30 μM p-AmBleb. The αCatenin TS indicator showed a marked increase in relaxation ratio, whereas αCatenin-EGFP control showed little or no change. Each dot in the scatter plot indicates the fluorescence intensity of each cell (**A, C**). The shaded areas indicate the 95% CIs (**B**). Statistical analysis was performed using one-way ANOVA followed by the Scheffé post hoc test (**B**); ns: $p > 0.05$, *: $p < 0.05$, **: $p < 0.01$, ***: $p < 0.001$. Asterisks indicate statistical significance at 2, 6, 10, and 14 min compared to 0 min.

Supplementary Figure 4

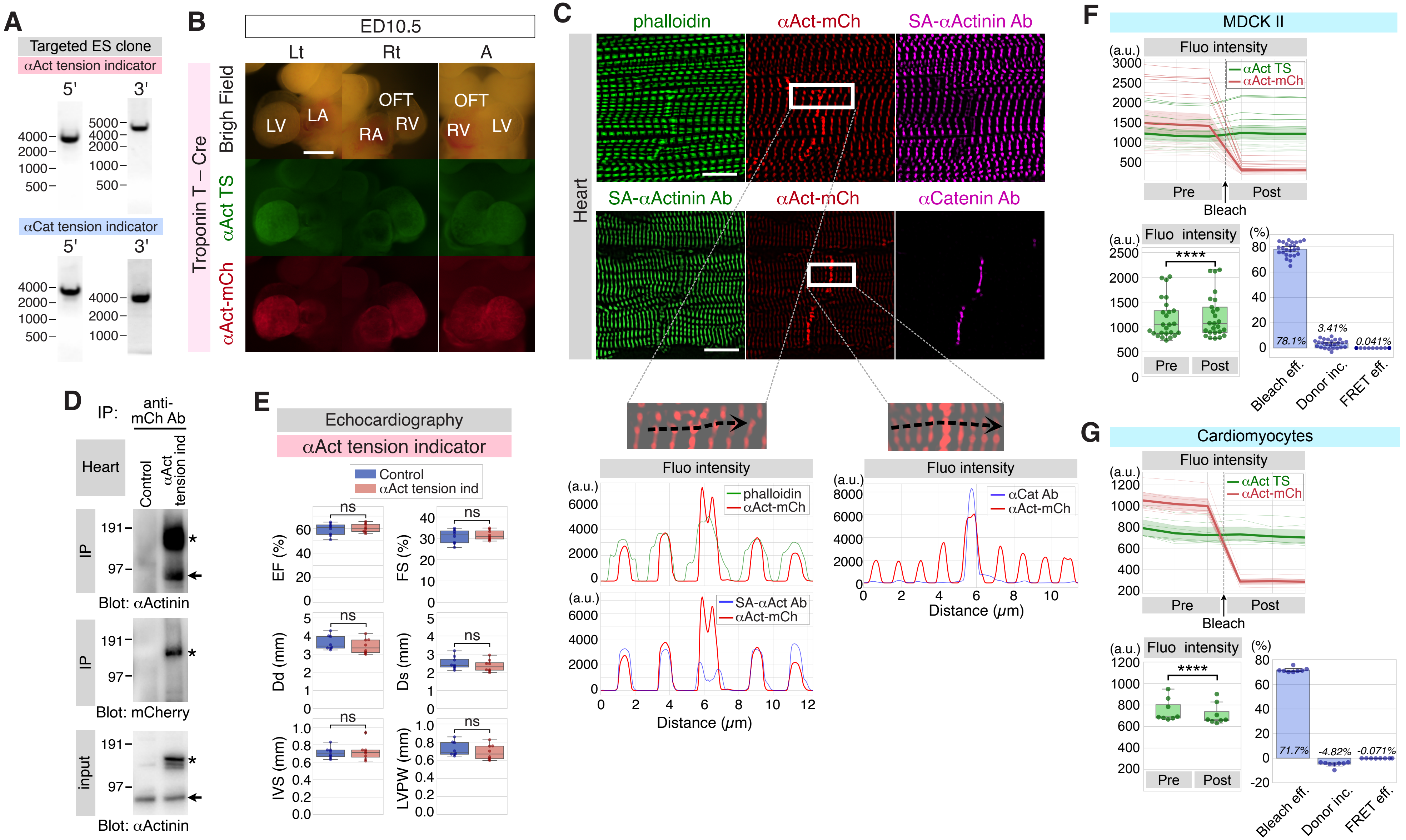

Supplementary Figure 4: **The αActinin tension indicator behaves in the same manner as the endogenous αActinin. A,** Genomic PCR analysis confirming the correct targeting of the αActinin and αCatenin tension indicator cassettes at both 5' and 3' junctions. The specific clones used for mouse generation are shown. **B,** Fluorescence stereomicroscopy of mouse embryo with the αActinin tension indicator crossed with Troponin T-Cre (ED10.5). Scale bar: 400 μm. ED, embryonic day; LV, left ventricle; LA, left atrium; RV, right ventricle; RA, right atrium; OFT, outflow tract. **C,** Superresolution fluorescence microscopy of immunostaining of heart tissue sections from αActinin tension indicator mice. αActinin-mCherry indicates red fluorescence from the transgene without antibody staining. Scale bar: 50 μm. The top panels are a tissue section of the heart fixed with 4% paraformaldehyde (PFA), while the bottom panels are a tissue section of the heart fixed with 100% methanol, resulting in different textures and appearances. Quantification of the fluorescence intensities of αActinin-mCherry and phalloidin staining or anti-SA-αActinin immunostaining along the indicated arrows is also shown. Note that αActinin-mCherry is properly localized at the z-disc or cell junction in cardiomyocytes. **D,** Western blot analysis of heart lysates immunoprecipitated with either anti-mCherry antibody or control IgG. Note that the αActinin tension indicator interacts with endogenous αActinin and the αActinin tension indicator itself. **E,** Echocardiographic data of αActinin tension indicator mice at 4 weeks of age (n = 8 mice per group). Box plots show the median (center line), upper and lower quartiles (box limits), and 1.5× interquartile range (whiskers). There was no change in cardiac function (EF and FS), ventricular diameter (Dd and Ds) or ventricular thickness (IVS and LVPWD). **E, F,** Quantification of intermolecular FRET efficiency in MDCKII cells in vitro (n = 25 cells) (**F**) and in cardiomyocytes in vivo (n = 8 cells) (**G**) expressing the αActinin tension indicator. A weak FRET signal was detected only under conditions of artificially high transient overexpression in MDCKII cells, whereas no detectable FRET was observed at physiological expression levels (**F**). Cardiomyocytes from αActinin tension indicator mice likewise exhibited no measurable FRET (**G**). These results indicate that intermolecular FRET between the TS module and mCherry is negligible under both physiological and in vivo conditions. Statistical analyses were performed using an independent t-test. Box plots show the median (center line), upper and lower quartiles (box limits), and 1.5× interquartile range (whiskers); ns: $p > 0.05$, **** $p < 0.0001$.

Supplementary Figure 4: **The αActinin tension indicator behaves in the same manner as the endogenous αActinin.** **A,** Genomic PCR analysis confirming the correct targeting of the αActinin and αCatenin tension indicator cassettes at both 5' and 3' junctions. The specific clones used for mouse generation are shown. **B,** Fluorescence stereomicroscopy of mouse embryo with the αActinin tension indicator crossed with Troponin T-Cre (ED10.5). Scale bar: 400 μm. ED, embryonic day; LV, left ventricle; LA, left atrium; RV, right ventricle; RA, right atrium; OFT, outflow tract. **C,** Superresolution fluorescence microscopy of immunostaining of heart tissue sections from αActinin tension indicator mice**.** αActinin-mCherry indicates red fluorescence from the transgene without antibody staining. Scale bar: 50 μm. The top panels are a tissue section of the heart fixed with 4% paraformaldehyde (PFA), while the bottom panels are a tissue section of the heart fixed with 100% methanol, resulting in different textures and appearances. Quantification of the fluorescence intensities of αActinin-mCherry and phalloidin staining or anti-SA-αActinin immunostaining along the indicated arrows is also shown. Note that αActinin-mCherry is properly localized at the z-disc or cell junction in cardiomyocytes. **D,** Western blot analysis of heart lysates immunoprecipitated with either anti-mCherry antibody or control IgG. Note that the αActinin tension indicator interacts with endogenous αActinin and the αActinin tension indicator itself. **E,** Echocardiographic data of αActinin tension indicator mice at 4 weeks of age (n = 8 mice per group). There was no change in cardiac function (EF and FS), ventricular diameter (Dd and Ds) or ventricular thickness (IVS and LVPWD). **F, G,** Quantification of intermolecular FRET efficiency in MDCKII cells in vitro (n = 25 cells) (**F**) and in cardiomyocytes in vivo (n = 8 cells) (**G**) expressing the αActinin tension indicator. A weak FRET signal was detected only under conditions of artificially high transient overexpression in MDCKII cells, whereas no detectable FRET was observed at physiological expression levels (**F**). Cardiomyocytes from αActinin tension indicator mice likewise exhibited no measurable FRET (**G**). These results indicate that intermolecular FRET between the TS module and mCherry is negligible under both physiological and in vivo conditions. Statistical analyses were performed using an independent t-test. Box plots show the median (center line), upper and lower quartiles (box limits), and 1.5× interquartile range (whiskers); ns: $p > 0.05$, **** $p < 0.0001$.

Supplementary Figure 5

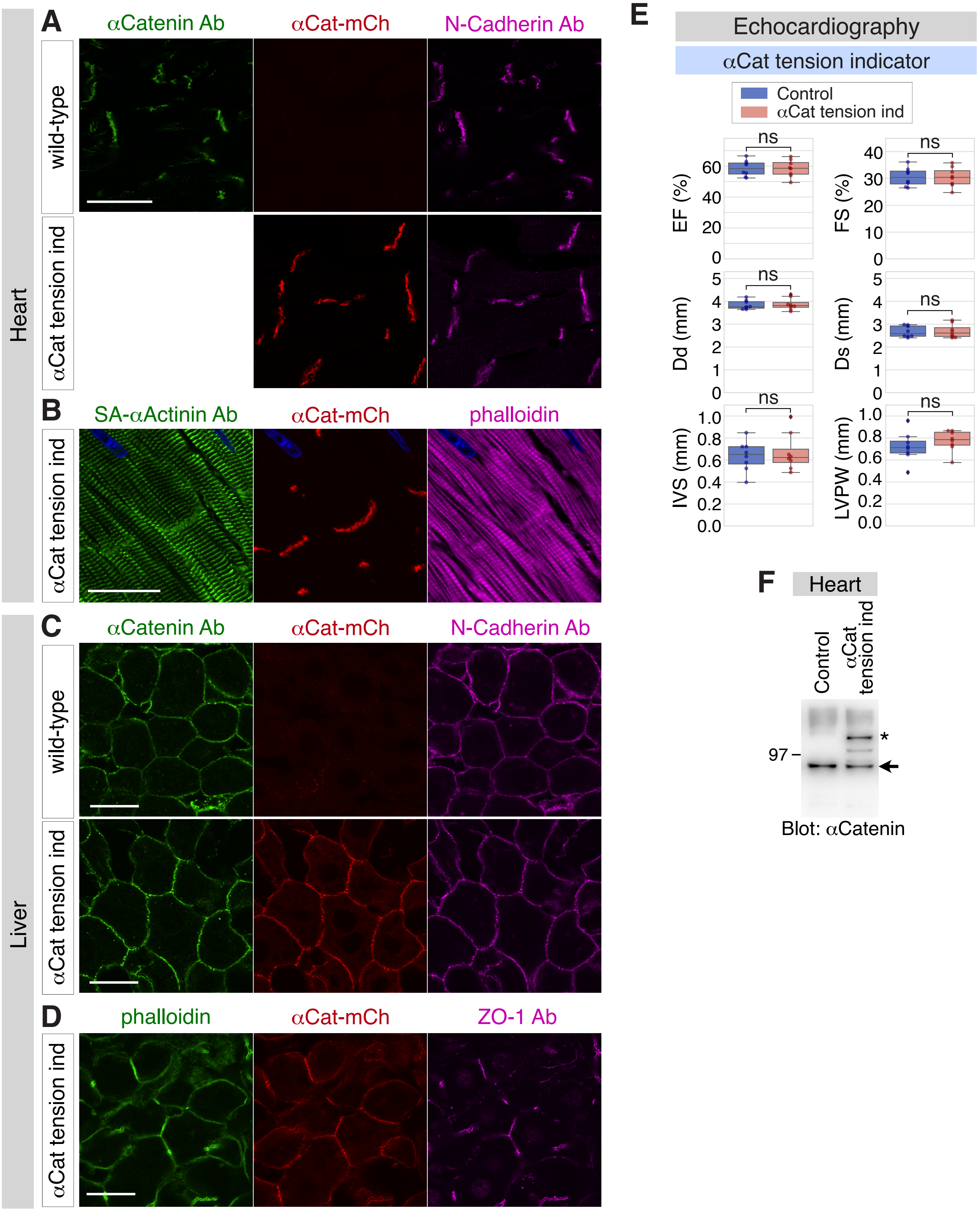

Supplementary Figure 5: **The αCatenin tension indicator is properly localized and has no harmful effects. A, B,** Confocal fluorescence microscopy of immunostaining of heart tissue sections from either wild-type or αCatenin tension indicator mice. The sections were immunostained with each indicated antibody or stained with phalloidin. Scale bar: 20 μm. **C, D,** Confocal fluorescence microscopy of immunostaining of liver tissue sections from either wild-type or αCatenin tension indicator mice. The sections were immunostained with each indicated antibody. Note that αCatenin-mCherry is properly localized at cell-cell junctions in the liver; however, the distribution of N-Cadherin or αCatenin does not match the distribution of ZO-1, which specifically labels tight junctions surrounding bile canaliculi in the liver. Scale bar: 20 μm. **E,** Echocardiographic data of αCatenin tension indicator mice at 4 weeks of age (n = 8 mice per group). There was no change in cardiac function (EF and FS), ventricular diameter (Dd and Ds) or ventricular thickness (IVS and LVPWD). Statistical analyses were performed using an independent t-test; ns: $p > 0.05$. Box plots show the median (center line), upper and lower quartiles (box limits), and 1.5× interquartile range (whiskers). **F,** Western blot analysis of heart lysates from control and αCatenin tension indicator mice. The αCatenin tension indicator (*) and endogenous αCatenin (arrow) are indicated.

Supplementary Figure 5: **The αCatenin tension indicator is properly localized and has no harmful effects.** **A, B,** Confocal fluorescence microscopy of immunostaining of heart tissue sections from either wild-type or αCatenin tension indicator mice. The sections were immunostained with each indicated antibody or stained with phalloidin. Scale bar: 20 *μ*m. **C, D,** Confocal fluorescence microscopy of immunostaining of liver tissue sections from either wild-type or αCatenin tension indicator mice. The sections were immunostained with each indicated antibody. Note that αCatenin-mCherry is properly localized at cell-cell junctions in the liver; however, the distribution of N-Cadherin or αCatenin does not match the distribution of ZO-1, which specifically labels tight junctions surrounding bile canaliculi in the liver. Scale bar: 20 *μ*m. **E,** Echocardiographic data of αCatenin tension indicator mice at 4 weeks of age (n = 8 mice per group). There was no change in cardiac function (EF and FS), ventricular diameter (Dd and Ds) or ventricular thickness (IVS and LVPWD). Statistical analyses were performed using an independent t-test; ns: $p > 0.05$. Box plots show the median (center line), upper and lower quartiles (box limits), and 1.5× interquartile range (whiskers). **F,** Western blot analysis of heart lysates from control and αCatenin tension indicator mice. The αCatenin tension indicator (*) and endogenous αCatenin (arrow) are indicated.

Supplementary Movie Legends

Supplementary Movie 1.

Time-lapse fluorescence microscopy images of lamellipodia from an NIH3T3 cell expressing the αActinin tension indicator. Images of the relaxation ratio, which was calculated by dividing the green fluorescence of αActinin-TS by the red fluorescence of αActinin-mCherry. Image acquisition rate: 1 frame / min. Video frame rate: 8 frames / sec. Scale bar: 10 μm.

Supplementary Movie 2.

Time-lapse fluorescence microscopy images of the filopodia of an NIH3T3 cell expressing the αActinin tension indicator. Images of the tension ratio, which was calculated by dividing the red fluorescence of αActinin-mCherry by the green fluorescence of αActinin-TS. Image acquisition rate: 1 frame / min. Video frame rate: 8 frames / sec. Scale bar: 10 μm.

Supplementary Movie 3.

Time-lapse superresolution fluorescence microscopy images of the protrusion of an NIH3T3 cell expressing the αCatenin tension indicator. Merged images of αCatenin TS and αCatenin-mCherry. Image acquisition rate: 1 frame / min. Video frame rate: 8 frames / sec. Scale bar: 5 μm.

Supplementary Movie 4.

Time-lapse superresolution fluorescence microscopy images of the cell margin of an NIH3T3 cell expressing the αCatenin tension indicator. Merged images of αCatenin TS and αCatenin-mCherry. Image acquisition rate: 1 frame / min. Video frame rate: 8 frames / sec. Scale bar: 5 μm.

Supplementary Movie 5.

Time-lapse super-resolution fluorescence microscopy images of an isolated cardiomyocyte expressing the αActinin tension indicator before and after the addition of blebbistatin. Merged images of αActinin TS and αActinin-mCherry. Image acquisition rate: 1 frame / min. Video frame rate: 8 frames / sec. Scale bar: 20 μm.

Supplementary Movie 6.

Time-lapse superresolution fluorescence microscopy images of a cardiomyocyte expressing the αActinin tension indicator. Magnified images of the region surrounded by the rectangle in Fig. 4D.

Note that the color of the merged image of αActinin-TS and αActinin-mCherry shifted toward green over time after the addition of blebbistatin. Image acquisition rate: 1 frame / min. Video frame rate: 8 frames / sec. Scale bar: 2 μm.

Original blot/gel images

Figure 1

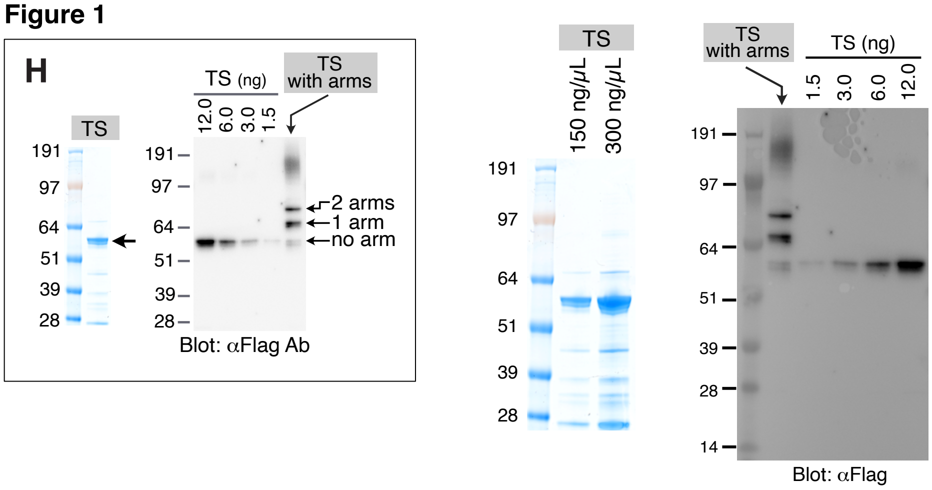

Supplementary Figure 4

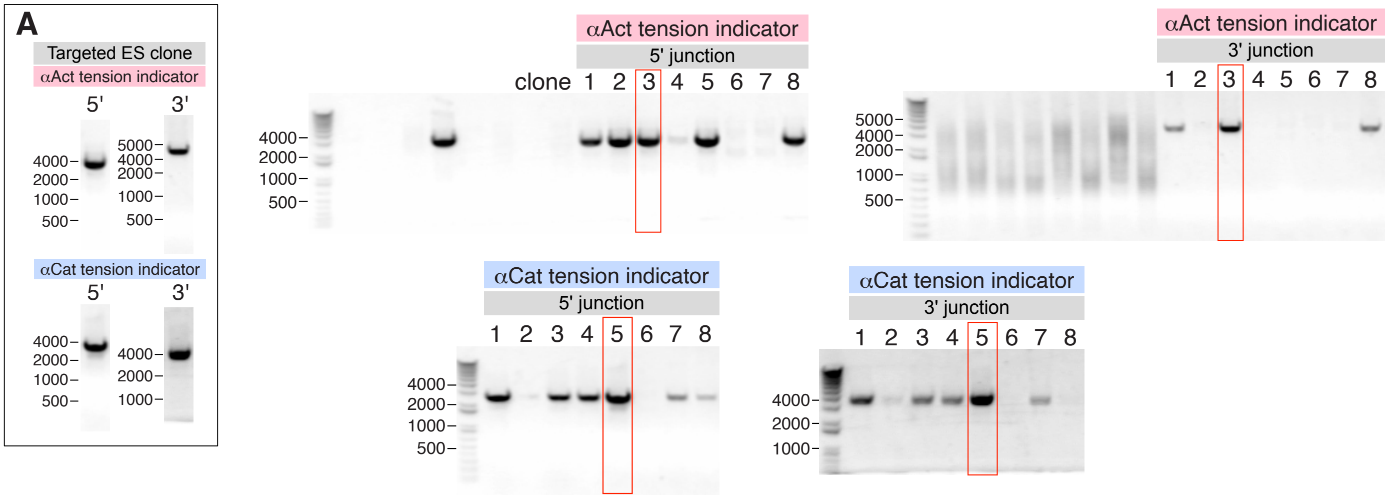

Supplementary Figure 4

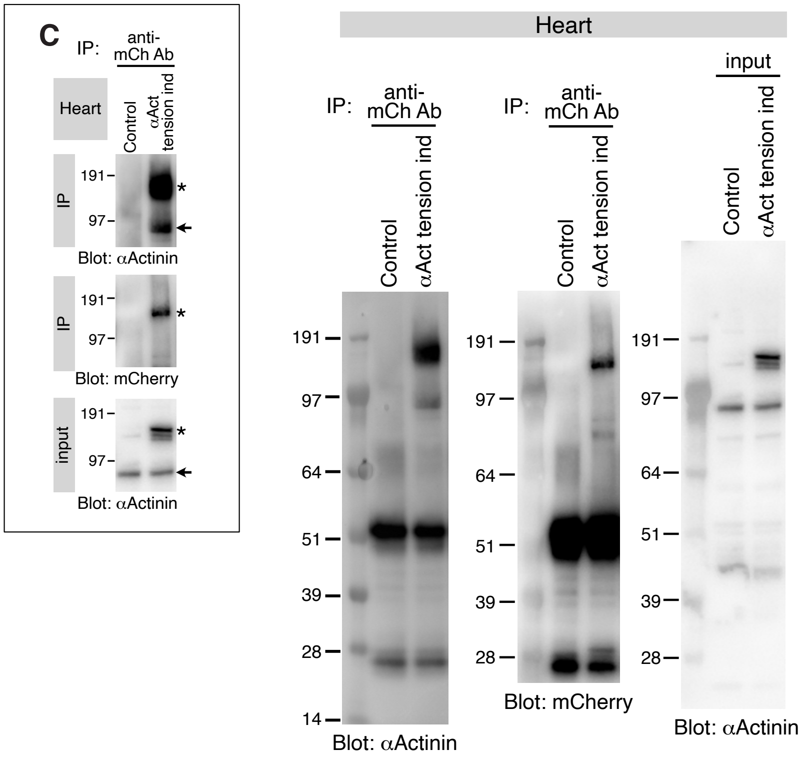

Supplementary Figure 5

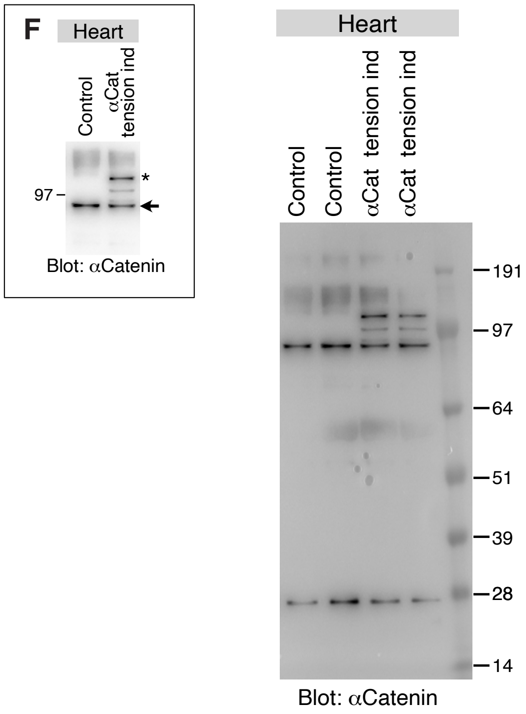

Supplement: Supplementary file 2 — Supplementary Information [file 42003_2026_9746_MOESM2_ESM.pdf]
