# Supplementary material for: Molecular tension indicators reveal unexpectedly complex regulation of tension in live mouse organs
Source: Commun Biol. 2026 Feb 19;9:455. doi: 10.1038/s42003-026-09746-0 (PMC13031782; doi:10.1038/s42003-026-09746-0)
Supplement: Supplementary file 3 — Description of Additional Supplementary Files [file 42003_2026_9746_MOESM3_ESM.pdf]

Description of Additional Supplementary Files

File name: Supplementary Data 1
Description: Excel file containing the source data behind the graphs in the paper.

File name: Supplementary Data 2
Description: Plain text file containing plasmid DNA sequences.

File name: Supplementary Movie 1
Description: Lamellipodia dynamics: αActinin tension indicator.

File name: Supplementary Movie 2
Description: Filopodia dynamics: αActinin tension indicator.

File name: Supplementary Movie 3
Description: Cell protrusion: αCatenin tension indicator.

File name: Supplementary Movie 4
Description: Cell margin: αCatenin tension indicator.

File name: Supplementary Movie 5
Description: Cardiomyocyte expressing αActinin tension indicator.

File name: Supplementary Movie 6
Description: Magnified view of the cardiomyocyte shown in Supplementary Movie 5.
