# Supplementary material for: Molecular tension indicators reveal unexpectedly complex regulation of tension in live mouse organs
Source: Commun Biol. 2026 Feb 19;9:455. doi: 10.1038/s42003-026-09746-0 (PMC13031782; doi:10.1038/s42003-026-09746-0)
Supplement: Supplementary file 12 — Reporting Summary [file 42003_2026_9746_MOESM12_ESM.pdf]

nature portfolio

Corresponding author(s): Maretoshi Hirai

Last updated by author(s): Dec 8, 2025

Reporting Summary

Nature Portfolio wishes to improve the reproducibility of the work that we publish. This form provides structure for consistency and transparency in reporting. For further information on Nature Portfolio policies, see our Editorial Policies and the Editorial Policy Checklist.

Statistics

For all statistical analyses, confirm that the following items are present in the figure legend, table legend, main text, or Methods section.

n/a	Confirmed	
☐	☒	The exact sample size (*n*) for each experimental group/condition, given as a discrete number and unit of measurement
☐	☒	A statement on whether measurements were taken from distinct samples or whether the same sample was measured repeatedly
☐	☒	The statistical test(s) used AND whether they are one- or two-sided<br>*Only common tests should be described solely by name; describe more complex techniques in the Methods section.*
☐	☒	A description of all covariates tested
☐	☒	A description of any assumptions or corrections, such as tests of normality and adjustment for multiple comparisons
☐	☒	A full description of the statistical parameters including central tendency (e.g. means) or other basic estimates (e.g. regression coefficient) AND variation (e.g. standard deviation) or associated estimates of uncertainty (e.g. confidence intervals)
☐	☒	For null hypothesis testing, the test statistic (e.g. *F*, *t*, *r*) with confidence intervals, effect sizes, degrees of freedom and *P* value noted<br>*Give P values as exact values whenever suitable.*
☒	☐	For Bayesian analysis, information on the choice of priors and Markov chain Monte Carlo settings
☒	☐	For hierarchical and complex designs, identification of the appropriate level for tests and full reporting of outcomes
☒	☐	Estimates of effect sizes (e.g. Cohen's *d*, Pearson's *r*), indicating how they were calculated

Our web collection on statistics for biologists contains articles on many of the points above.

Software and code

Policy information about availability of computer code

Data collection	Fiji (ImageJ2: 2.16.0, ImageJ1: 1.54p) was used for fluorescence image processing. Confocal images were acquired using EZ-C1 (Nikon), FLUOVIEW FV3000 (Olympus), and Fusion (Andor Technology) software. C-trap optical tweezer data were collected and visualized using Lakeview (Lumicks).
Data analysis	Fiji (ImageJ2: 2.16.0, ImageJ1: 1.54p) and Imaris 10.2 (Oxford Instruments) were used for image processing and 3D reconstruction. SRRF-Stream super-resolution images were processed with Fusion software and corrected for chromatic shifts using Chromagon. C-trap HDF5 datasets were imported with Python 3.11.12 using h5py and analyzed using Python's scientific ecosystem, including pandas 2.3.3, NumPy 1.26.4, SciPy 1.15.2, Matplotlib 3.10.3, Seaborn 0.13.2, and scikit-learn 1.5.2. Statistical analyses were performed in Python using SciPy, pandas, and NumPy. Schematic diagrams were prepared using Affinity Designer 2.5.7.

For manuscripts utilizing custom algorithms or software that are central to the research but not yet described in published literature, software must be made available to editors and reviewers. We strongly encourage code deposition in a community repository (e.g. GitHub). See the Nature Portfolio guidelines for submitting code & software for further information.

Data

Policy information about availability of data

All manuscripts must include a data availability statement. This statement should provide the following information, where applicable:

- Accession codes, unique identifiers, or web links for publicly available datasets
- A description of any restrictions on data availability
- For clinical datasets or third party data, please ensure that the statement adheres to our policy

All numerical source data supporting the findings of this study are provided in the Supplementary Information.

Research involving human participants, their data, or biological material

Policy information about studies with human participants or human data. See also policy information about sex, gender (identity/presentation), and sexual orientation and race, ethnicity and racism.

Reporting on sex and gender	n/a
Reporting on race, ethnicity, or other socially relevant groupings	n/a
Population characteristics	n/a
Recruitment	n/a
Ethics oversight	n/a

Note that full information on the approval of the study protocol must also be provided in the manuscript.

Field-specific reporting

Please select the one below that is the best fit for your research. If you are not sure, read the appropriate sections before making your selection.

☒ Life sciences ☐ Behavioural & social sciences ☐ Ecological, evolutionary & environmental sciences

For a reference copy of the document with all sections, see nature.com/documents/nr-reporting-summary-flat.pdf

Life sciences study design

All studies must disclose on these points even when the disclosure is negative.

Sample size	No statistical methods were used to predetermine sample size. Sample sizes are consistent with those used in the field and were sufficient to detect the reported effects.
Data exclusions	No data were excluded from the analyses.
Replication	All experiments were replicated independently with similar results. The number of biological replicates is described in the figure.
Randomization	Samples were not randomized because experimental groups (e.g., control vs mutant mice) were defined by genotype. Within each group, animals were processed identically.
Blinding	Investigators were not blinded to experimental groups because genotype and fluorophore expression were visually distinguishable during imaging.

Reporting for specific materials, systems and methods

We require information from authors about some types of materials, experimental systems and methods used in many studies. Here, indicate whether each material, system or method listed is relevant to your study. If you are not sure if a list item applies to your research, read the appropriate section before selecting a response.

Materials & experimental systems

n/a	Involved in the study	
☐	☒	Antibodies
☐	☒	Eukaryotic cell lines
☒	☐	Palaeontology and archaeology
☐	☒	Animals and other organisms
☒	☐	Clinical data
☒	☐	Dual use research of concern
☒	☐	Plants

Methods

n/a	Involved in the study	
☒	☐	ChIP-seq
☒	☐	Flow cytometry
☒	☐	MRI-based neuroimaging

Antibodies

Antibodies used	Primary antibodies used for immunofluorescence were as follows: anti-αActinin (Sarcomeric) mouse monoclonal (1:200, clone EA53, Sigma-Aldrich, cat. A7732), anti-αCatenin rabbit polyclonal (1:200, Sigma-Aldrich, cat. C2081), anti-N-Cadherin mouse monoclonal (1:200, clone 32, BD Bioscience, cat. 610920), and anti-ZO-1 rabbit polyclonal (1:200, ThermoFisher, cat. 40-2200). Secondary antibodies for immunofluorescence were Alexa Fluor 488, 546, or 647 conjugated anti-rabbit, anti-mouse, or anti-rat IgG (ThermoFisher). Phalloidin staining was performed using Alexa Fluor 647 Phalloidin (ThermoFisher, cat. A22287) or phalloidin-iFluor 488 conjugate (Cayman Chemical, cat. 20549), and nuclei were counterstained with Hoechst 33342 (1 mg/mL, 1:50).<br>For immunoprecipitation and Western blotting, protein lysates were precleared with protein G agarose conjugated to normal rabbit IgG (Santa Cruz, cat. sc2027), followed by immunoprecipitation using an anti-mCherry antibody (Abcam, cat. ab167453). Primary antibodies for Western blotting were anti-mCherry polyclonal (1:1000, Abcam, cat. ab167453) and anti-αActinin polyclonal (Proteintech, cat. 11313-2-AP). HRP-conjugated rabbit or mouse IgG secondary antibodies (1:3000; R&D Systems, cat. HAF008 and HAF007, respectively) were used for detection.
Validation	All primary antibodies used in this study are commercially available and have been validated by the manufacturers for immunofluorescence, immunoprecipitation, or Western blotting in mouse tissues. Validation information is available on each supplier’s website. In addition, αActinin, αCatenin, N-Cadherin, and ZO-1 exhibited their well-established subcellular localization patterns in cardiomyocytes or hepatocytes, consistent with previous literature and supporting antibody specificity.

Eukaryotic cell lines

Policy information about cell lines and Sex and Gender in Research

Cell line source(s)	MDCKII and NIH3T3 cell lines were obtained from established laboratory stocks. Both cell lines are widely used and not derived from human participants or primary tissues.
Authentication	The MDCKII and NIH3T3 cell lines used in this study were not independently authenticated by STR profiling; however, their morphology and growth characteristics were consistent with published descriptions.
Mycoplasma contamination	The cell lines were not tested for mycoplasma contamination.
Commonly misidentified lines<br>(See ICLAC register)	MDCKII and NIH3T3 cell lines are not listed as commonly misidentified cell lines in the ICLAC register.

Animals and other research organisms

Policy information about studies involving animals; ARRIVE guidelines recommended for reporting animal research, and Sex and Gender in Research

Laboratory animals	All mouse experiments were performed in accordance with institutional guidelines at Kansai Medical University. Gene-targeted mice expressing either the αActinin or αCatenin tension indicator were generated using EGR-05 ES cells (derived from B6/129 F1). Correctly targeted ES cell clones were identified by PCR and injected into 8-cell-stage ICR embryos to produce chimeric mice, and germline transmission was confirmed by genotyping. The resulting gene-targeted mice were maintained by backcrossing with ICR mice. For experiments requiring ubiquitous expression, gene-targeted mice were crossed with Ayu-Cre mice. Additional mouse lines used in this study included Ayu-Cre mice (kindly provided by Dr. K. Yamamura, Kumamoto University) and Troponin T-Cre mice (The Jackson Laboratory, strain #024240). All mice were maintained under standard housing conditions.
Wild animals	This study did not involve wild animals.
Reporting on sex	Sex was not considered as an experimental variable in the study design. For echocardiography experiments, male and female mice were selected such that each genotype group contained an equal number of males and females. No sex-based analyses were performed.
Field-collected samples	This study did not involve field-collected samples.
Ethics oversight	All animal procedures were approved by and conducted under the oversight of the Institutional Animal Care and Use Committee at

Ethics oversight	Kansai Medical University, in accordance with institutional and national guidelines.

Note that full information on the approval of the study protocol must also be provided in the manuscript.

Plants

Seed stocks	n/a
Novel plant genotypes	n/a
Authentication	n/a
